# Transfer of musculoskeletal care from paediatric to adult services for patients with cerebral palsy and chronic neuromuscular conditions: Identifying the unmet need

**DOI:** 10.1016/j.hctj.2024.100078

**Published:** 2024-11-02

**Authors:** Bhushan Sagade, Connor Thorn, Portia Ross, Catherine May, Evan Davies, Darius Rad, Caroline Edwards, Alexander Aarvold

**Affiliations:** University Hospitals Southampton NHS Foundation Trust, Tremona Road, Southampton SO16 6YD, UK

**Keywords:** Transfer services, Cerebral Palsy, Transfer of care, Musculo-skeletal care

## Abstract

**Introduction:**

Children with cerebral palsy (CP) are treated by well-co-ordinated multi-disciplinary neuromuscular teams. With a comprehensive multidisciplinary team, co-ordinating the transfer to adult care is a challenge. Orthopaedic care becomes fragmented as patients transfer from paediatric orthopaedic surgeons with training in multi-joint neuromuscular conditions (NMCs), to adult orthopaedic surgeons where this expertise rarely exists. Orthopaedic and spinal problems are a major unmet health need in this population. There is a lack of research in the literature regarding availability and access to orthopaedic services post transferring, which this study aimed to address.

**Methods:**

This was a cross-sectional study conducted at an NHS referral centre for specialist paediatric services to evaluate the existing transfer program. A 10-item questionnaire was developed by senior clinicians for patients with CP or NMCs and their families to answer. It focused on three domains: Availability of a transfer of care plan, access to healthcare services and satisfaction with the services.

**Results:**

There were 39 responses from patients, or their families received between December 2021 to April 2022. Patients were aged between 17 and 28 years at the time of answering. Availability of a transfer of care plan was poor, at only 20.5 % (8/39). Sixty-nine percent (27/39) stated there was not an orthopaedic surgeon overseeing their bone and joint health and a further 33.3 % (13/39) reported lack of supervision from a physiotherapist. Fifty-six percent (22/39) of respondents reported a decline in musculoskeletal health. Those that did receive ongoing orthopaedic care reported high rates of satisfaction.

**Conclusion:**

Our study has shown that the transfer of care for patients with CP and NMCs continues to remain poor, contrary to national guidelines, with lack of access to adequate musculoskeletal healthcare services after transfer to adulthood. The unmet needs of patients with CP or NMCs with orthopaedic and spinal pathologies are higher than previously reported. This area has been critically understudied, but this manuscript has highlighted an urgent need to improve and reform transfer practises, to fulfil the current deficit.

## Introduction

Children with Cerebral Palsy (CP) and Chronic Neuromuscular conditions (NMCs) are treated within the UK by well-co-ordinated multi-disciplinary teams. Multiple health professionals are invariably involved during the childhood years, including specialist paediatricians, sub-specialist orthopaedic surgeons, neurologists, physiotherapists, community paediatricians, orthotists, occupational therapists, specialist nurses and the General Practitioner (GP) amongst others.^1^ Many patients with CP and NMCs have life expectancies near those in healthy populations and even in those with the most severe disease, 60 % are expected to reach adulthood.^2^ It follows that the vast majority of these patients will have to transfer from paediatric to adult services; all of this at a time when they are experiencing a significant change in their lives socially, learning the responsibilities of adulthood, perhaps looking for work or planning further education or independent living. The familiar healthcare team that the patients and their families have known throughout their childhood changes completely as they transition to adulthood. With such a comprehensive multi-disciplinary team being involved in patients’ care, co-ordinating this transfer of care is difficult. This is invariably a difficult time both for the patient and their families, and a logistical challenge for the health care system.

Bone and joint problems are amongst the highest unmet health needs in this patient population,^3^ with major impact on function and quality of life.^4^, ^5^ Problems include fragility fractures, spasticity, contractures, scoliosis, chronic joint dislocation, and early onset arthritis. A specific difficulty in musculoskeletal care in CP and NMCs as the child transitions to adulthood, is the cessation of the involvement of the paediatric orthopaedic surgeon, who possesses training in multi joint neuromuscular conditions. This speciality barely exists in adult orthopaedic surgery in the UK, whereby sub specialisation is into specific body regions or joints. Thus, the musculoskeletal care can become fragmented. Potential communication obstacles between the different sub-specialties, alongside being discharged from the community paediatrician, can result in a sub-optimal service being provided to patients.

Transition of neuromuscular patients from childhood into adult care has been actively discussed since the 1980’s.^6^ Progress has been made in creating guidelines that serve as a benchmark for the standard of care that is expected to be delivered to these young adults with special needs, including National Institute for Health and Care Excellence (NICE) Guidelines.^7^ However, achieving these standards remains an elusive reality.

NICE guidelines^7^ suggest this transition should be actively considered when the patient reaches teenage years, with clear pathways in place involving GPs and the paediatric team. Similar recommendations are made in the 2018 National Confidential Enquiry into Patient Outcome and Death report ‘Each and Every Need’.^8^ This aims to ensure the patient has access to services in adulthood with appropriate CP training. There is little published literature on this topic and so it is difficult to determine whether these guidelines are followed and if patients are successfully able to access the services that they require and that are recommended. Furthermore, the specialist skillset of the paediatric orthopaedic surgeon in care of children with CP and NMCs rarely exists in the world of orthopaedic surgery in adulthood, whereby joint specific expertise is the norm. The small amount of published literature suggests that the UK is failing to provide a comprehensive and satisfactory service,^9^, ^10^, ^11^ with only 6 % of patients reported to receive a transition plan.^11^ This is despite almost half of patients having ongoing needs in their limb bones and joints and a quarter having ongoing needs relating to their spine. A similar picture is also seen internationally.^12^, ^13^ Previous literature has studied practices to improve the outcome of transitioning^7^, ^11^ but, to the best of our knowledge, there have been no studies on issues with availability and access to healthcare post transitioning.

A formal transition programme was implemented in our NHS trust in 2012, titled ‘Ready, Steady, Go’.^14^ This programme provides guidelines on the transition process with the intention of delivering high-quality transition for young persons with chronic/lifelong conditions. These include the availability of a key individual, a documented plan along with a health passport and it also has an emphasis on patient and parent education for the move to adult care. This is based upon the Care Quality Commission’s recommendations and is the standard that should be met for all patients under our care. Auditing the specifics of transition for something like epilepsy, whereby there are experts in epilepsy in both paediatric and adult care settings, is fairly straightforward. This is more complex in musculoskeletal medicine, whereby care tends to become fragmented amongst adult orthopaedic surgeons with joint specific expertise (e.g., feet or knees or hips).

This study was therefore conducted to audit the existing transfer of care practice at our NHS trust against the ‘Ready, Steady, Go’ guidelines, with an emphasis on musculoskeletal aspects (limbs and spine) in patients with CP and NMCs, plus family satisfaction with the transfer process.

## Methods

This was a service review of the transfer of care at our institution - a tertiary referral centre for specialist paediatric services. A defined snapshot of four months was chosen, conducted between December 2021 and April 2022. A questionnaire was used to assess the compliance of our paediatric orthopaedic and spine unit, against the standards set out in the ‘Ready, Steady, Go’ programme.^14^ An IRB approval was not required as this study is a service evaluation of an existing healthcare pathway. The study was conducted in accordance with the Declaration of Helsinki and after obtaining an informed consent from all the responders.

### Questionnaire

The parent/patient questionnaire was designed by consensus among healthcare professionals from multi-disciplines involved in the care for CP patients. The survey was checked for clarity and content by two physicians before being sent out to participants. There are ten questions, providing focus on the following domains of transfer of care: availability of a transfer of care plan, access to musculoskeletal healthcare services, satisfaction with the services received (Table 1). Satisfaction with services were rated on a Likert scale of 0 – 10.

**Table 1:** Questionnaire.

**Domain**	**Questionnaire Item**
Availability of transfer of care plan	Transfer of care plan received- Yes/No
Access to Healthcare Services	Who has taken over your general care (drooling, tone, seizures, PEG feeding, chest infections, urinary issues etc.) from your community/ hospital Paediatrician (child specialist)?
	Who has taken over your care for the management of muscle, bone, and joint issues?
	Who has taken over your care for the management of spine issues?
	Who has taken over your care for physiotherapy?
	How has your/ your child’s bone and joint health been after transfer of care from the children’s to the adult’s side?
Satisfaction with Healthcare Services	If you received a transfer of care plan, on a scale of 0 – 10, how happy are you with it? (0= very unhappy; 10= extremely happy)
	How happy are you with the care received from your current Orthopaedic doctor? (0= very unhappy; 10= extremely happy)
	How happy are you with the care received from your current spine doctor? (0= very unhappy; 10= extremely happy)
	Any Comments or Suggestions

### Sample

In order to establish whether a transfer of care had occurred, the care pathway of a cohort of patients was reviewed. Patients included had a diagnosis of CP or other NMCs, were aged >16 years, previously treated within paediatric orthopaedic department in our trust, were now discharged from the children’s services. Families in whom there were known safeguarding issues were not included, in order to avoid potential confounding factors in family engagement with services (though this is of course quite common and adds further complexity to the difficulties of transitioning/transfer).

### Data collection

Patient’s electronic records were accessed to obtain demographics, including age, gender and gross motor function classification system (GMFCS) levels. GMFCS is a descriptor of the level of severity of physical disability, with Level 1 being essentially no physical disability and Level 5 being full disability with no independent or voluntary movement or control. This has a bearing on the services that are likely to be required through to adulthood. The electronic patient records were also reviewed for the presence or absence of a documented transition plan. Patients or their families were contacted via a phone call to explain the purpose of the service review. With verbal consent from the designated caregiver (or patient if they had capacity), an online survey was sent via email, including formal written informed consent prior to commencing the service evaluation survey.

### Data analysis

Data was analysed using Excel (Version 16.49, Microsoft 2021) and described using averages and standard deviations (SD) as appropriate. T test and Chi Square tests were used for comparison between groups, with a focus on those who had a transfer plan and those that did not. P values of < 0.05 were considered significant.

## Results

A total of 63 patients / families were contacted for invitation to take part in the survey. These were all within the defined inclusion criteria described. Ten families declined to participate, thus, a total of 53 patients were available for participation in this service evaluation.

There were 39 responses received (74 %), of which, 90 % (35) were given by parents and 10 % (4) were given by patients themselves. The average age (SD) of the respondent patients was 21.3 years (± 2.8), ranging from 17 – 28 years. There were 23 males and 16 females. The neurological diagnosis was CP in 32 patients and seven had other NMCs such as spina bifida, muscular dystrophy or a genetic syndrome. The distribution of GMFCS level is given in Table 2, with the majority being GMFCS 5.

**Table 2:** GMFCS Levels.

**Gross Motor Function Classification System (GMFCS)**	**Number of patients (n=39)**
1	1
2	0
3	5
4	7
5	19
Not CP*	7

* Seven patients were not CP and hence were not classified by GMFCS Levels

### Availability of a transfer of care plan

A formally documented transfer of care plan was identified for eight out of 39 patients (21 %), with a further seven (18 %) who had a letter to their GP stating possible referrals to an appropriate adult orthopaedic colleague in the future if necessary. Chi-square test revealed no difference in GMFCS levels between those who received a plan vs. those who did not (p=0.59).

Nine respondents (23 %) reported receiving a transfer of care plan whereas 30 respondents (77 %) reported they did not. Interestingly, seven of the nine who claimed that they had a documented transfer of care plan in their health records, did not actually have one after all. There were six families who denied having a transfer of care plan, despite there being one formally documented in their notes. Thus overall, 20.5 % patients had a documented transfer of care plan in their electronic health records.

### Access to musculoskeletal services

**Fig. 1:**
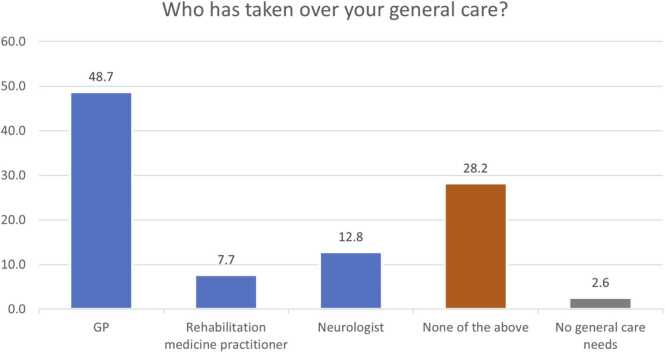
Transfer of general care needs.

**Fig. 2:**
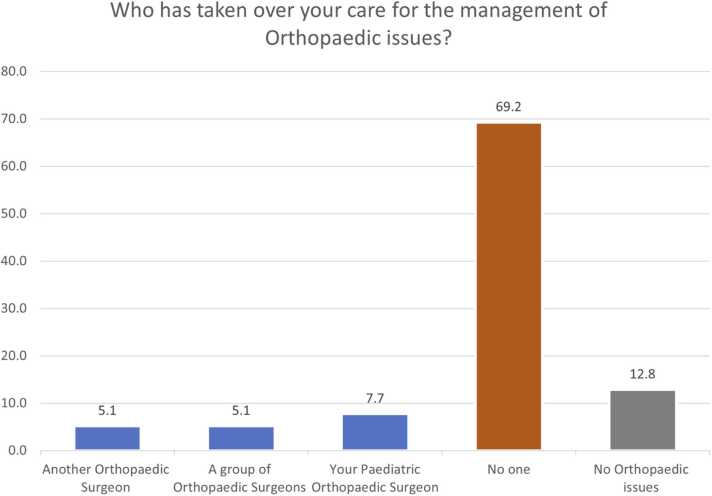
Transfer of limb musculoskeletal care.

**Fig. 3:**
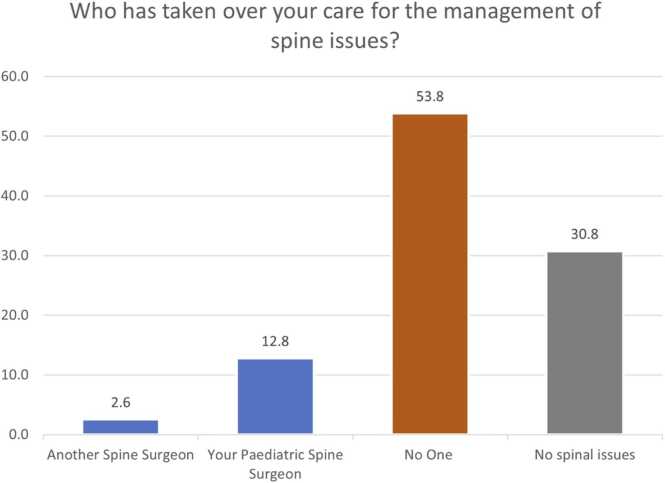
Transfer of Spinal care.

**Fig. 4:**
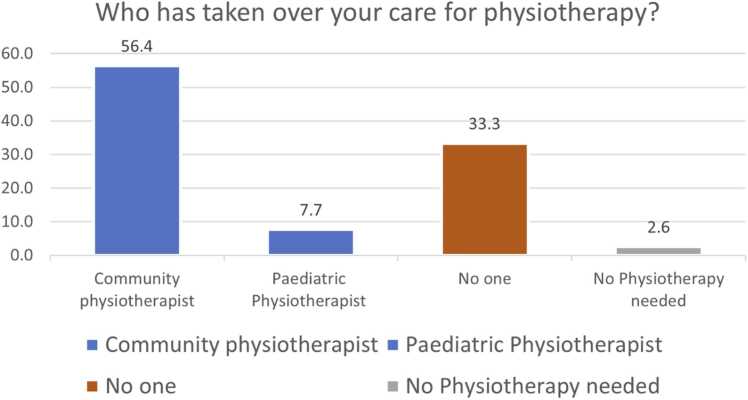
Transfer of care for physiotherapy.

**Fig. 5:**
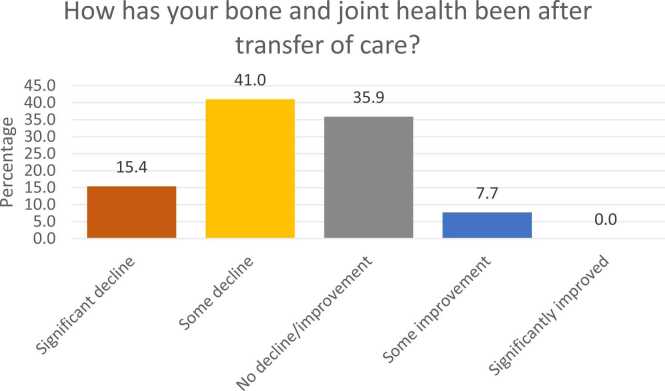
Musculoskeletal health after transfer to adult services.

There were 12.8 % (5/39), 30.8 % (12/39), and 2.6 % (1/39) of patients who reported no ongoing orthopaedic, spinal or physiotherapy needs respectively. In terms of transfer of general care needs, 49 % (19/39) respondents reported that their overall care was taken over by their GP, 12.8 % (5/39) by a Neurologist, and 7.7 % (3/39) by a Rehabilitation medicine practitioner. Just over 28 % (11/39) reported no one taking over general care needs and 2.6 % (1/39) reported no general care needs. Sixty-nine percent (27/39) reported lack of orthopaedic surgeon overseeing their bone and joint health, 54 % (21/39) of respondents denied availability of spinal surgeon, and 33 % (13/39) of patients reported lack of supervision by any physiotherapist (Fig. 1, Fig. 2, Fig. 3, Fig. 4). This is alongside a background of 56 % (22/39) patients who reported a decline in their musculo-skeletal health (Fig. 5).

There were 87 % (34/39) patients with continuing orthopaedic needs of which 69 % (27/39) remained unmet. The proportion of unmet needs is 0.79. Similarly, 69 % (27/39) patients needed continuing spinal care of which 54 % (21/39) remains unmet. The proportion of unmet needs for spinal care is 0.78. The average score of satisfaction with the transfer of care plan was 4/10 on a Likert scale (SD 3.6; Range 0–10).

### Satisfaction with services received

Patients who received a transfer of care plan had average satisfaction scores of 4/10 as compared to 1.9/10 for those who did not (p= 0.09). Average score of satisfaction for patients who continued to receive orthopaedic or spinal supervision was 8.5/10 as compared to 1.5/10 for those who did not (p< 0.001). Patients who continued to receive orthopaedic and spinal supervision and treatment had average scores of satisfactions of 7.9/10 (SD 1.5; Range 5–10) and 8.7/10 (SD 0.9; Range 8–10) respectively. There was no statistically significant difference in the score of satisfaction between the patients requiring orthopaedic versus spinal needs (p = 0.44).

## Discussion

### Overview

This is an evaluation of musculoskeletal transfer of care from childhood to adulthood for patients with CP and NMCs. It is an area that is critically understudied. This study has identified an alarming failure in the implementation of transfer of care in this group of patients. Shockingly, a majority of these vulnerable patients with CP and NMCs (79 %) had had no formal transfer of care plan. At the same time, an alarming proportion (56 %) described a decline in their musculoskeletal health after discharge from paediatric services. The need is clearly there.

### Disparity in receipt of transfer of care plan

The disparity between the families who reported receiving a transfer of care plan vs. those who did not, shows the lack of understanding about the process of transfer of care in the families, perhaps also, a gap in communication between the healthcare professionals (HCPs) and the patients, or lack of clarity as to what a transfer of care plan in the patient charts should read like. Despite having an established programme running in our hospital,^14^ 79.5 % patients did not receive a transfer of care plan, indicating lack of adherence to the guidelines. On comparison with literature, 6 % reported receiving a transition plan in the study by Ryan et al.^11^ while the number was 20.5 % in our study.

### Proportion of unmet needs

The survey highlights the unmet orthopaedic and spinal needs of patients with CP and NMCs that have been transferred to adult services. Twenty-eight percent patients denied any oversight of their care. The lack of continuing care by respective professionals (Fig. 1, Fig. 2, Fig. 3, Fig. 4) reflects on the musculoskeletal health of these individuals.

We identified a greater percentage of needs for orthopaedic and spinal issues (87 % and 69 %) as compared to Solanke et al.^10^ (41.5 % and 24.7 %) with a consequently higher proportion of unmet needs. Winger et al. have identified musculoskeletal concerns in 30 % of their study cohort, which in their opinion was underrepresented due to high volume of other concerns.

### Reasons for lack of a transfer of care plan

The lack of a transfer of care plan despite having a transition programme running in our hospital could be due to the following reasons: Lack of Time, Lack of Awareness, Lack of a healthcare team into which to transfer the child. The NHS is currently under tremendous pressure and there are long waiting times for outpatient appointments. Tackling this is likely to be a higher political priority than expanding current transitional care pathways, which would assume secondary importance. This underlies the importance of this study in highlighting this unmet need.

There is also lack of awareness in the treating team regarding experiences that their patients/families may have to go through after transferring to adult services. Lastly, transitioning is talked about less since there is no clear healthcare team in the adult services that can potentially take over the musculoskeletal care of these patients, as we have previously alluded to in the introduction.

### Discussion of our results

The results of our study lead us to believe that the support for young people with complex needs is lacking, alongside a deficit of adult healthcare providers. This issue is very specific to the orthopaedic side of care for these patients. The lack of appropriate services with HCPs having condition specific knowledge has been highlighted in a previous study.^15^ General care was overseen by GP’s alone in 49 % of patients in adulthood. GPs in the UK are already overstretched, with a high workload and a staffing shortage. There is an expectation for GPs to pick up the care of these complex cases that was previously provided by a comprehensive paediatric team. This is neither practicable nor realistic and alternatives need to be explored.

### Role of Keyworkers

A recent study from a centre providing transitioning care to adults with complex disabilities including CP has identified the need for care coordination as the most prevalent healthcare concern.^16^ Hence, keyworkers play a crucial role in coordination of care for patients with complex health needs.^17^, ^18^ The NHS long-term plan includes a commitment of providing all children and young people with learning disability and autism, a keyworker by 2023/24.^19^ If this commitment could be extended to young adults with CP, perhaps much of the deficit in care identified here would be remedied.

### Strategies for bringing change

Our study shows that there is extensive scope for improvement in the transition services, including the transfer of care. Short term goals for improvement must begin with commitment to provide better transition services to young people with CP and NMCs. Patients and HCPs alike, should be educated about the importance of transitioning. Educating patients can involve distribution of information leaflets and videos to patients at appointments and display of banners in clinics. Organising telephone clinics about transition involving a support worker or a doctor may also be of use. Importance of transition should be ingrained into the minds of HCPs by including it in their training programme. A module on transition practices can also be included in NHS eLearning courses for staff that are involved in care of these children. This can build on those provided by, for example, Health Education England.

In the medium term, the scope of keyworkers should be expanded to support children with CP and other NMCs with complex needs. This should be complimented by setting up age banded clinics or transition clinics involving both children and adults with CP and NMCs. Longer term reforms can target development of an adult neuromuscular speciality, as this would increase the number of HCPs available to address needs of adults with CP and NMCs. Evaluation and feedback about the service should continue to identify any shortcomings and rectify them.

### Limitations

To the best of our knowledge, ours is the only study which has included patient satisfaction, however, it is limited by a small sample size coupled with response and recall biases. We have not taken into consideration the living location and the socio-economic status of these patients which may act as potential confounding factors. Majority of the patients were GMFCS IV-V. It may indicate their complex health needs and hence, the difficult transfer period. The difficulties of accessing healthcare were adversely affected by the COVID-19 pandemic which would further adversely impact patient experiences. The questionnaire did not undergo rigorous development process and has not been validated. However, it does ask questions specific to musculoskeletal care, for which we are not aware of any pre-existing satisfactory equivalent. Furthermore, rehabilitation need is a hypothetical construct and there is no set standard against which we can compare the questionnaire to establish criterion or construct validity.^20^

## Conclusion

In conclusion, the provision of care after transfer of children with CP and NMCs to adult services continues to remain poor with lack of access to adequate healthcare services after transitioning. The unmet needs of orthopaedic and spinal patients are higher than reported before. Reassuringly, those patients who did continue to receive care after the transfer of their care were very satisfied by it. There is a need and scope for significant reforms in practices so that patients do not feel abandoned after discharge from paediatric clinics. Our centre’s experience is likely shared by others around the country. Further studies are needed pertaining to knowledge, beliefs, and practices in adult orthopaedic clinics to identify obstacles to treating adults with CP after the transfer of their care.

## Ethics Approval and consent to participate

As this is a service evaluation ethics approval was not necessary.

Informed consent was obtained from patients or their families verbally via telephone call

## Funding

No sources of financial support.

## CRediT authorship contribution statement

**Alexander Aarvold:** Writing – review & editing, Supervision, Conceptualization. **Caroline Edwards:** Writing – review & editing, Supervision, Conceptualization. **Darius Rad:** Writing – review & editing, Supervision, Conceptualization. **Evan Davies:** Writing – review & editing, Supervision, Conceptualization. **Catherine May:** Writing – review & editing, Methodology, Data curation, Conceptualization. **Portia Ross:** Writing – review & editing, Writing – original draft, Methodology, Data curation, Conceptualization. **Connor Thorn:** Writing – review & editing, Writing – original draft, Data curation. **Bhushan Sagade:** Writing – review & editing, Writing – original draft, Methodology, Formal analysis, Data curation, Conceptualization.

## Declaration of Competing Interest

The authors declare that they have no known competing financial interests or personal relationships that could have appeared to influence the work reported in this paper.

## Data Availability

No data was used for the research described in the article.
